# Towards a Chinese Conception of Adolescent Development in a Migration Context

**DOI:** 10.1100/tsw.2007.66

**Published:** 2007-04-30

**Authors:** Ching-man Lam

**Affiliations:** Department of Social Work, The Chinese University of Hong Kong, Shatin, N.T., Hong Kong

**Keywords:** adolescent development, Chinese culture, migration

## Abstract

Although there are many well-known theories of adolescent development in the West, there is a notable lack of theory with empirical support to understand the process and outcome of Chinese adolescent development. This paper attempts to advance a Chinese conception of adolescent development in a migration context. A qualitative study approach was used to explore the experiences and views of 19 Chinese-Canadian adolescents from Hong Kong and ten of their parents. The findings indicate that parents and adolescents co-construct the dominant theme of “covert parental control” in the adolescent development process, and the concept of “self in relational networks” as the adolescent development outcome. Based on the developmental experiences of these Chinese-Canadian adolescents, a culturally sensitive model of Chinese adolescent development is proposed. This model incorporates culture and migration as two essential components of the framework for a theory regarding Chinese adolescent development. It acknowledges the experience of Chinese-Canadian immigrants, takes account of the participants' personal meanings, and incorporates the indigenous Chinese cultural concepts of bao (reciprocity), guan (guidance), and guanxi (relationship).

## INTRODUCTION

This paper aims to advance a framework in which the developmental process and outcome of adolescence in a migration context can be understood. Theoretical and empirical reviews reveal that there is a lack of cultural perspective in the existing adolescent development theories and a lack of cultural diversity in the data on adolescent development[1,2]. Traditionally, theories of adolescent development have adopted an etic perspective, which emphasizes universals among human beings. Our contemporary knowledge about adolescent behavior and the existing theories of adolescent development are embedded within a Western cultural code[3,4,5,6], and the study of human behavior is dominated by North American theories[7,8,9]. With the recognition that human nature has no existence independent of culture[10], and that meanings are embedded and constructed within a social and historic context[11], the traditional quantitative paradigm that rests on racist, classicist, and ethnocentric forms of relations is now seen as inadequate[12,13,14]. The recurring, conceptual flaws and methodological shortcomings in research with racially and ethnically diverse youths[9], and the lack of cultural perspective in our current adolescent development theories is a central issue[2,15]. A theoretical framework that incorporates culture and migration as two essential categories in searching for a theory of adolescent development in immigrant families has been proposed[5]. The proposed framework serves as a guide for a qualitative study to review critically the adolescent experience, and to create culture- and context-specific meanings in relation to adolescent development. Based on the findings, this paper attempts to advance a Chinese conception of adolescent development in a migration context.

The Chinese conception of adolescent development is guided, first, by the assumption that adolescent development is a social construct and that culture is at the core of the construction[10,16]. Culture, which is submerged beneath consciousness, functions as a system of ideas, beliefs, and values for interpreting human experiences, and as the filter through which understanding passes[17]. It is also a code through which we make sense of reality, experience, and behavior. In order to understand adolescent development, the significant impacts of culture and values on the construction of the category of adolescent development must be addressed.

Second, adolescent development in immigrant families is a complex and multifaceted issue. The developmental experience of adolescence is not universal, but is context specific. As is well known, expectations and behaviors are culturally defined and are socially constructed. Being immigrants in a new and foreign country, Chinese immigrant families are exposed to two very different, but somehow equally important, cultures. The specific characteristics of immigrants, and the sociopolitical situation of the home and the host culture, have important implications for meaning making and the adaptation process. The cultural diversity and the complex and distinct context of migration bring particular meaning to the issue of adolescent development.

Third, the model is based on the notion that there is a dynamic interplay and an interactional process between culture and migration in immigrant families that affects the developmental process of adolescents. Chinese immigrant families experience a developmental adjustment process during the adolescent stage that is different from either the traditional Chinese model or the dominant Western model. Given these assumptions, it is expected that in Chinese immigrant families, there is a particular transformation pattern and a particular meaning of adolescent development that is co-constructed by the host culture, the home culture, and the migration context.

The proposed Chinese conception of adolescent development is based on the findings of a qualitative study. A large volume of data has been generated from this study[18,19]. The foci for discussion in this article are the developmental process and its outcome among Chinese immigrant adolescents. The contextual factors, which are the migration context and the dominant Chinese cultural theme of Chinese familism, and the three culturally indigenous themes — the notion of relationships (guanxi), the concept of reciprocity (bao), and the idea of guidance (guan) — will be elaborated in an attempt to advance a Chinese conception of adolescent development in a migration context.

## METHODS

This study uses the qualitative research method because the qualitative paradigm is suited to the perspective guiding the research (a constructivist-interpretive view, which contends that reality is open to multiple constructions), the research problem (exploratory and contextual in nature), and the audience (a minority group in a migration context).

The study is based on the grounded theory approach of Glaser and Strauss[20,21]. Following the “constructivist paradigm”[11,22], this study adopts the view that reality is the result of social processes accepted as normal in a specific context[11] and that what is seen as real is a construction in the minds of individuals[22]. As such, the meaning-making process must be interpreted in a cultural context[16], and human phenomena are best studied with no manipulation by the inquirer and no a priori views on the outcome[22].

As the objective of this research is to understand the experience of a particular subgroup of immigrant families in depth, a homogeneous group of participants was selected. All participants were first-generation immigrants from Hong Kong and were living in the Toronto area, with differing lengths of residence in Canada (6–12 years). Adolescent participants interviewed were in the later stage of adolescence (16–21 years of age). Nineteen adolescents from 16 families (in the case of three families, two adolescents were interviewed) and ten of their parents participated in this research. Of the adolescent participants, ten were male and nine were female. Of the parents, eight were mothers and two were fathers. The characteristics of the participants are listed in the participant profile (Table 1).

**TABLE 1**

| | Name of Adolescent* | Gender | Age | Grade** | Name of Parent | Age | Education | Occupation | Length of Residence in Canada |
|---|---|---|---|---|---|---|---|---|---|
| 1 | Andrew | M | 17 | Grade 12 | Mrs. A | 48 | High school | Housewife | 11 years |
| | Billy | M | 20 | OAC | | | | | |
| 2 | Charles | M | 19 | Year 1 (University) | Mrs. C | 46 | College | Lab. technician | 12 years |
| | Doris | F | 17 | Grade 11 | | | | | |
| 3 | Edward | M | 18 | Grade 11 | Mrs. E | 49 | High school | Clerk | 7.5 years |
| 4 | Flora | F | 17 | Grade 12 | Mrs. F | 42 | High school | Housewife | 7 years |
| 5 | George | M | 20 | Year 1 (University) | Mrs. G | 46 | High school | Accounting clerk | 7 years |
| 6 | Helen | F | 17 | Grade 12 | —*** | — | — | — | 6 years |
| 7 | Irene | F | 19 | Year 1 (University) | — | — | — | — | 9.5 years |
| 8 | Jenny | F | 16 | Grade 11 | Mrs. J | 58 | College | Housewife | 8+ years |
| 9 | Kelvin | M | 17 | Grade 12 | — | — | — | — | 6.5 years |
| 10 | Larry | M | 20 | Year 2 (University) | — | — | — | — | 10 Years |
| 11 | Mary | F | 17 | Grade 12 | — | — | — | — | 6+ years |
| 12 | Nelson | M | 17 | Grade 11 | Mrs. N | 44 | High school | Housewife | 6 years |
| 13 | Oliveria | F | 21 | Year 3 (University) | — | — | — | — | 8 years |
| 14 | Paul | M | 17 | Grade 11 | Mrs. P | 42 | High school | Mortgage sales manager | 8 years |
| | Queenie | F | 16 | Grade 10 | | | | | |
| 15 | Rose | F | 18 | OAC | Mr. R | 50 | University | Property manager | 9 years |
| 16 | Stephen | M | 17 | Grade 12 | Mr. S | 54 | College | Businessman | 7 years |

*Their nicknames are given according to the order of the interview sequence and their gender.

**All the adolescent participants are students.

***Unable to participate.

### The Interviewing Process

Open-ended, individual interviews were conducted following an interviewing guide (the detailed interview guide is available from the author). Questions dealt with the meaning of adolescence, the variables that influence the adolescent developmental process, patterns of development, dominant themes in parent-adolescent relationships, ideological and cultural values with regard to parenthood, and contextual and cultural factors affecting adolescent development process and outcome.

Usually, the interview began with the question, “Can you describe for me your experiences in the past few years?” This is an example of a “grand tour” question[23] intended to help participants talk from a place in which they are “experts” (i.e., drawing on their own experience). The advantages of this kind of open-ended interviewing format are that it is “interviewee-oriented” and it “encourages creativity”[24]. The primary focus during the interviews was on understanding the voices, feelings, perceptions, and subjective experiences of the participants. The researcher became immersed in the participants’ stories, listening intently to share the participants’ experience, clarifying and probing for information so that understanding would be comprehensive and detailed.

### Data Analysis

All interviews were audiotaped and the tapes were transcribed verbatim. The tape-recorded interviews were then analyzed to reveal emergent themes and to establish various categories, by using the methods of qualitative data analyses[25,26,27]. Nudist[28], a computer-assisted qualitative data analysis software package, was employed to organize files, code data, and retrieve selected passages by category.

## RESULTS

### Process of Adolescent Development — Covert Parental Control

Mrs. A (nicknames are given to each research participant according to the order of the interview sequence and their gender) shared her views on parenting. Although her friends advised her to allow her rebellious son to learn from his mistakes, she firmly stated that she found such an approach nonsensical and she believes that parents should prevent their children from making mistakes. Mrs. F also said repeatedly, “It is the responsibility of parents to stop children from going on the wrong path.” Mrs. P echoed these sentiments, stating that the Western way of allowing children to learn by mistakes is “a waste of time” and “harmful to the children”. This view was not confined to these three parents. All the parents interviewed shared a view that the role of parents is to prevent children from wrongdoing, to help children to “distinguish between right and wrong”, and to direct children “on the right road”. The findings showed that a dominant theme in parents’ narratives is, “we are held responsible for our children’s development”.

The parent interviewees revealed that their major concern is how to be responsible parents who are able to guide their children in a Western country. The parents were aware that they are now living in another culture where it is less acceptable to be overbearing; therefore, they use subtle means to convey expectations and implicit rules to their children. Throughout the interviews, the subtle nature of parent-adolescent interaction and the indirect nature of parental influence were apparent.

According to the adolescents, their parents were “skillful in giving instruction”, “they convey the message or tell me their opinion indirectly”, they “brainwash us” and “are successful in molding us”. The parents, in turn, revealed that, although they had high expectations of their children, they used subtle means to achieve their goals. For example, the parents seldom expressed their concerns and hopes to their children as direct expectations; they conveyed their feelings in subtle ways. They seldom “enforced rules”; they expressed worry and fear and left their children to devise “self-initiated curfews”. They seldom instructed their children directly, but used their relationship with their children to, in one mother’s words, “help them make the right choice.” They practiced filial piety themselves to set a good model for their children. They celebrated the Chinese festivals and took every opportunity in daily life to implant Chinese cultural traditions. They adopted a watchful attitude to protect their children from perceived negative influences. They explained their views and preferences, and encouraged their children to see the consequences of their decisions. They approached matters rationally, using logic with their children, and, in this way, they conveyed their expectations. They practiced a “strategic authoritarian parenting style” of covert control.

What was the reaction of the adolescents? The data indicate that adolescents perceived their parents’ intentions and acted in accordance with them. All the adolescents realized that their parents were “trying to move [us] in a certain direction”, “there were lots of influences during our developmental process”, “they direct us to where they want us to go”, and if they had another idea, their parents would “take it [the idea] out”. In one of the adolescent’s wordings, “my parents are not really allowing me to decide” and “they will support their children if they do what the family wants them to do”. Although the adolescents expressed themselves in various ways, they all emphasized the guidance and control elements of the parenting process.

The adolescents appeared to have experienced a subtle psychological struggle, from mentally reframing, rationalizing, and justifying the process of covert control, to finally accepting and co-constructing it. During the course of the interviews, various emotional reactions, such as anger, discomfort, guilt, helplessness, and gratitude, were identified when adolescents addressed the “control” themes. However, the negative feelings were suppressed, restrained, reframed as “mutual goals”, and justified by the feeling of guilt and thankfulness. The majority of the adolescents accepted parental control and they maintained that, in their words, parental guidance “is like insurance to protect you against doing inappropriate things”, the curfew imposed by parents “pulled me away from heading towards the road of turning bad”, parents would never “point you to a dark road [the wrong way]”, and they “have a reason for doing so”. Some adolescents even verbally declared that loyalty to their family is a must and they cared about their family’s reputation; they consider the feelings of their parents, and respect parental wishes and authority when making decisions.

The findings indicate that the dominant theme underlying the process of adolescent development in the Chinese immigrant families is “covert parental control”. “Covert control” in this context refers to a form of parenting characterized by parental guidance, family teaching, coaching, and monitoring. Parents, through “tact” and “skills”, successfully influence and guide their children. Adolescents, on the other hand, are very much concerned with filial piety and subordinate their personal concerns to their parents’ conventional demands. They share family beliefs, values, and thinking, and are obedient to their parents. The data suggest that a dominant theme of “covert parental control” has been co-constructed by parents and adolescents during the adolescent process in the Hong Kong Chinese immigrant families.

## Outcome of Adolescent Development — Self in Relational Networks

The open-ended question, “What is the desired outcome of adolescent development?” attempted to identify the themes in parents’ and adolescents’ construction of the desired outcomes of adolescent development. According to the descriptions of the participants, the desired outcomes of adolescent development include being a good person, being a good son/daughter, being a self-reliant person who honors family, and being a mature person.

Participants identified being a good person as the first desired outcome. They shared a common definition of a good person as “one who will not harm others”; “don’t do something that is for your own benefit, but that causes others to suffer”; “the bottom line is not to hurt others”; and “be able to face yourself and the sky (God)”.

Being a good son/daughter is the second theme. The criteria for being a good son/daughter, according to parents, includes “giving money to parents as a sign of filial pity”; “considering your family, bringing glory to your family and your descendants”; and “taking care of parents even after you marry”. Like their parents, adolescents consider “paying back their family”, “taking good care of their parents”, and “spending time with them” as criteria for being a good son/daughter.

“Be a self-reliant person” is the third theme. Adolescents mentioned that being financially independent and having a stable job are basic criteria. All adolescents agreed that, in order to attain the goal of self-reliance, one should study hard and be reliable and responsible. The participants connected self-reliance with responsibility, reliability, having good prospects, and studying hard. It is interesting to note that although the participants talk about self-reliance, their understanding of self-reliance is in relation to others and the ultimate goal of self-reliance is not for the “self”, but for the parents; self-reliance brings honor to the family.

“Being a mature person” is the fourth theme. First of all, “responsibility” is regarded as the essential quality of a mature person. As reflected in the adolescents’ comments, if you are a responsible person, you “not only consider yourself, you think for others”. When a mature person does something, “they consider the consequences first”, “think about others first”, “would consider what others would do and how others would react”, and “should be very considerate”. The narratives of the participants indicated their view that a responsible person is defined as a considerate, “other-oriented” person.

The narratives of the participants demonstrated that the expected adolescent outcomes center on issues of responsibility for, care of, and inclusion of other people. According to them, a mature self is one who is able to develop a web of well-suited, harmonious, appropriate, socially acceptable, and appropriately positioned social networks of relationships. The data suggest that a self that is appropriately positioned in the relational networks (self in relational networks) is the desired developmental outcome of Chinese adolescents.

## DISCUSSION

The dynamic interplay between two contextual factors, culture and migration, as well as the three indigenous concepts, bao (reciprocity), guan (guidance) and guanxi (relationship), help us to take a broader perspective in our attempt to understand the Chinese parents’ and adolescents’ construction of adolescent development processes and outcomes.

### The Migration Context — Sacrifice and Guilt

The parents interviewed repeatedly initiated discussion of the effects of migration on their own lives and those of their children. They reported that they kept asking themselves questions such as “Was my decision to migrate really the right choice for my children?” and “Was my sacrifice a worthwhile one?” “Sacrifice and guilt” are the meanings parents and adolescents attach to migration. The participants construct their migration experience as a sacrifice, a sacrifice on the part of the parents for the sake of a better future for their children.

Several factors account for this construction. The first is the background of the participants. As a result of the points system of immigration selection, people who have migrated to Canada from Hong Kong since the mid-1980s are educated, middle-class professionals[29,30,31,32,33,34]. The participants of this study also belonged to this group of immigrants who have sacrificed a great deal in migrating: the loss of a highly paid job, a familiar social and physical environment, and, most of all, a decline in social status and opportunities for career advancement.

Second, most of the parents revealed that they experienced discrimination and frustration when they tried to find work in Canada. According to them, prejudicial attitudes toward Chinese remain and a subtle form of racism exists; this further intensified their feeling of “sacrifice”. Prior to their migration, most of the parents were in full-time professional employment. However, during the period of residence in Canada, they encountered difficulties. Parents recalled the difficulties of finding jobs commensurate with their qualifications, and of persuading employers to recognize their previous work experience. In the interviews, it was clear that these immigrant parents experienced a drop in both income and employment status. Most of participants had experienced professional downgrading, “forced early retirement”, “change from dragon to snake”, or unemployment after migration. They felt that their talents and expertise were severely underutilized in Canada. Although they were psychologically prepared for the change, the reality was still not easy to accept. Consistent with findings on skilled migrants in other studies[35,36,37,38], Hong Kong immigrants revealed powerless feelings of being discriminated against and constrained by the disadvantaged factors, such as lack of Canadian experience, unequal opportunity, devaluation of foreign credential, and racism.

The impetus for their migration decision was to provide their children with better education for a better future. According to the parents’ accounts, their migration was motivated by the desire to “give the children a chance” and to “give their children the freedom to choose”. They regarded migration as a way of fulfilling their responsibility to their children. All participants stated that they would not have considered migration if they did not have children; migration is viewed as the parents’ sacrifice to give their children a better future.

If migration is seen as a sacrifice on the part of parents, then it is not surprising that their children express feelings of guilt and indebtedness. The adolescent participants have a sense of guilt for being the occasion of their parents’ sacrifice. They feel obligated to their family, and their sense of indebtedness becomes an internal control that encourages them to conform to parental expectation.

## The Cultural Context — Familistic Orientation

The participants’ discourses revealed supreme value placed on family, and their desire to maintain the well-being and the continuation of the family. Both adolescents and parents believe strongly in family solidarity, and emphasize the need for family cohesion and mutual help, family prosperity, and a strong sense of caring. Key themes in their discourse were responsibility towards the family, the interdependence of family members, and respect for and a sense of indebtedness to their parents. The findings not only demonstrate the central values of familism and the role of family among Hong Kong Chinese immigrants, they also demonstrate a result of migration, which I call “family-recentering”.

Migration is an uprooting experience in which immigrants are separated from their networks. The majority of the Hong Kong immigrants realized the need and expressed the wish to integrate into the host society, however, there were psychological blockages, cultural values and habits, and practical difficulties that made them retreat from the process of integration. Both parents and adolescents went through difficult times during their early adjustment period. Parents encountered discrimination and difficulties in employment. Adolescents described experiences of being discriminated against in high school. They revealed “problem fitting in” and “lacking of sense of sameness” when with Caucasian people. This state of alienation and isolation reinforced the primacy of the familial group, and the adoption of a suspicious and somewhat hostile attitude toward the host country. The family network, in contrast, gave them the help, support, and security they needed to settle in Canada. The experiences of economic segregation and social discrimination by the host society have been found to contribute to group cohesion[37]. As a result, the migrants immersed themselves in a network that was basically centered on their family and relatives. The familistic orientation thus was reinforced and intensified after migration.

The phenomenon of “family-recentering” has a direct impact on parent-child relationships. Traditionally, the Chinese place high value on family and children[39,40]. Given that the parent participants are a “self-selected” group of middle-class parents who are very concerned about the future of their children, most of them are, consequently, involved in their children’s socialization and make deliberate efforts to mold them. They spend time with their children and communicate with them. They strategically make use of every opportunity to influence them. They convey love, concern, and expectations to them. They closely observe and monitor their behavior. They attend to their children’s material, as well as emotional, needs. The majority of the adolescents revealed that they could feel the warmth and support of their parents. They appreciated what the family had done and the “sacrifice” they had made for them. The fact that the children continue to try to fulfill the wishes of their parents is probably the result of their parents' continuing to nurture, protect, and make sacrifices for them. In the families interviewed, there is mutual devotion and affective bonds that keep the family relatively intact. The cultural value of Chinese familism and characteristics of the Chinese parent-child relationship — a lifelong mutual dependence resulting from long-term cultivation and requiring deep commitment on both sides — had been reinforced in a migration context.

## The Indigenous Themes — Bao, Guan, and Guanxi

The account of the participants suggests that the migration context intensifies belief in three indigenous cultural themes: bao (reciprocity), guan (guidance), and guanxi (relationship). By incorporating these indigenous themes, we can understand how the "sacrifice and guilt" construction and the phenomenon of "family-recentering" developed, and how these indigenous concepts shape the immigrant adolescents' development process and outcomes.

Under the influence of Confucianism and Buddhism, the Chinese have developed an indigenous concept of reciprocity, bao. The belief underlying bao is that there is reciprocity of action between individuals[39]. When this concept is applied to the parent-child relationship, it implies that a child should be filial because he/she has received so much from their parents. In the interviews, the adolescents, although they might not express it directly, appeared to have a strong sense of indebtedness. Driven by a strong sense of obligation and guilt about the sacrifices made by their parents, as well as fully aware of the favors they have received from their family, the adolescents are determined to fulfill parental wishes and meet filial expectations. In the families interviewed, the concept of bao serves as the basis for parent-adolescent relationship and as a guide for their interaction. The migration context strengthens the cultural belief in bao and affected both immigrant parents and adolescents.

The Chinese concept of guan[41,42,43] further explains the dominant theme of "covert parental control" and the parenting behaviors. Throughout the past 2000 years, Chinese scholars have maintained a clear idea about the function of family education for the young and have placed strong emphasis on parental responsibility for instruction. In order to better understand Chinese parenting behavior, Chao[41,42] has identified an indigenous concept of guan, which includes aspects of training, teaching, governing, monitoring, controlling, and interfering, as a characteristic feature of Chinese socialization.

The findings of this study reflect that the migration context again reinforces the concept of guan in parenting behavior. The data indicated that the "we-they" separation continued to be the dominant theme. A cultural boundary between "we-Chinese" and "they-Westerners" and the explicit message that "we-Chinese" are not the same as "they-Westerners" were extremely apparent when talked about the expected outcome of adolescent development. Statements such as "Western parents are so and so", or "the Canadian society is so and so", or "they are not the same as us" were frequently heard during the interviews. The narrative accounts of parents reflected that they worried their children would be too "western" and would lose their "good Chinese traits". They also had reservations about the Western cultural norms and did not want their children to be the "true Westerner". During the interviews, parents kept revealing their disapproval of "Western culture" and expressed their "fear of Western influence". They were "concerned about the negative impacts of the host society". As a result, protecting children from the perceived negative influences of Western peers and the perceived negative impacts of Western society continued to be a major aim of the parent participants. In their efforts to counter the influences of Western culture, parents are very vigilant and watchful. The results indicate that the immigrant parents were determined to retain some basic elements of Chinese culture; they paid close attention to training and molding their children. The migration context, the experiences of social exclusion and discrimination, reinforces the concept of guan and results in stronger parental covert control.

Finally, the data inform us that the cultural belief in guanxi (relationships) is also intensified in the migration context. No one who has had firsthand experience of Chinese society could fail to note that

Chinese people are extremely sensitive to guanxi (relationships). Chinese people, in their daily lives, demonstrate impressive and sophisticated skills in guanxi building to establish personal resources.

Migration is an uprooting experience in which the migrants are forced to relinquish the social networks that have been established in their country of origin. These participants have learned that settling into the host society takes time. Their experiences have alerted them to the importance of restoring the disrupted social networks and “re-rooting” in the host society. Thus they have become active network builders in order to gain social support, which reduces their uncertainty about their situation, themselves, and their relationships. They make a deliberate effort to la (pull) quanxi, which means to establish or strengthen relationships with others when no pre-established relationship exists, or where their pre-established relationships are remote[44].

However, in a migration context, to la quanxi demands more effort with less reward, because quanxi building is usually based on shared attributes or long-term social quanxi (social exchange). Immigrant status, of course, handicaps the development of the quanxi wang (web of personal networks). Having difficulty to assimilate into the host society and to build up the western quanxi wang (network), Hong Kong migrants continue to be attached to their cultural group. The data revealed that kin and family members constituted their core social network. Friends and former colleagues from Hong Kong, and contacts made through attending Cantonese-speaking Chinese church, were other major networks. Hong Kong immigrants derived much support from participating in associations of Hong Kong Chinese[44]. The need for social support, the conditions of oppression experienced by migrants, and the experience of being uprooted and losing pre-established quanxi wang all are factors that reinforce their belief in the importance of network relationships and contribute to their determination to construct “self in relational networks”.

### Towards a Chinese Conception of Adolescent Development in a Migration Context

The dominant Western construction portrays the adolescent development process as a “separation-individuation process”[46,47]. The hallmark of adolescent development in this construction is the adolescents’ efforts to separate from their parents and so to achieve a sense of personal identity[46,48,49,50]. As to the desired outcome of adolescent development, Western thought places a high value on the idea of self-development, and hence the development of an autonomous and “firmly bounded” self is seen as the central task of adolescence[49,51]. Given the fact that Chinese and Western thinking originated from different philosophical, ideological, historical, and cultural contexts[52,53,54,55,56,57,58], the existing Western formulation of adolescent development might not be applicable to the Chinese. The results of this study indicate that immigrant parents and adolescents co-construct the dominant theme of “covert control” in the adolescent development process, and to develop a “self in relational networks” is the expected and ideal outcome of adolescent development.

Based on the experiences of the research participants, this paper attempts to advance a Chinese conception of adolescent development in a migration context. In the specific migration context, the indigenous Chinese concepts of guanxi (relationships), bao (reciprocity), and guan (guidance) are reinforced and intensified, and thus develop meanings and contribute themes to the adolescent development process and outcome. The interplay between these three themes and the way they affect the developmental process and outcome of Chinese adolescent development is shown in Fig. 1.

## CONCLUSIONS

The attempt to advance a Chinese conception of adolescent development does not intend to reinforce stereotypical assumptions, but aims to uphold the view that adolescent development is a social category and is a culturally defined issue. This view has significant theoretical and practical implications. In its

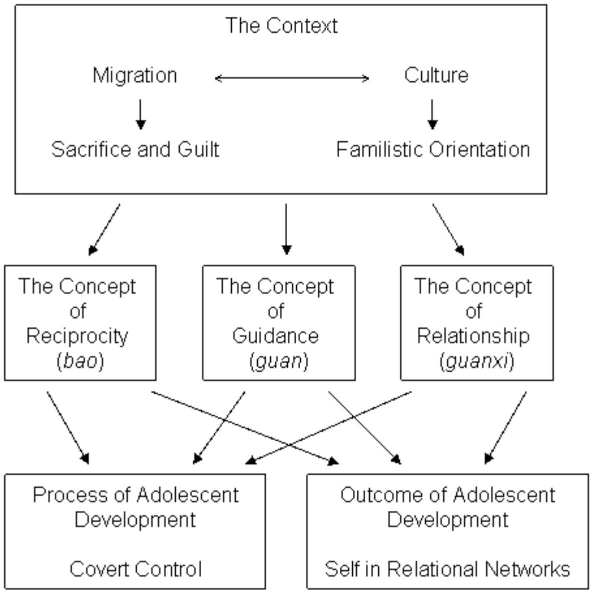

**FIGURE 1**. The Chinese conception of adolescent development in immigrant families.

theoretical implications, this study has been guided by the assumption that meanings are developed in a context. Individuals act and react on the basis of the meaning that they construct in a given situation. Using culturally shared vocabularies and structures, the Hong Kong Chinese immigrant parents and adolescents have constructed accounts to explain their experience of migration and of living in a new culture. As a cultural group, Hong Kong Chinese immigrants share a world view. Collectively, they interpret what goes on around them, assign motives, and develop shared understandings of social life. The way immigrants conceptualize their situation affects their interpretive, meaning-making, and value-generating activities. An understanding of this collective definition provides the background necessary to an understanding of the participants' narratives. The results illustrate the importance of context. This context-specific model of adolescent development, grounded in the findings of this study, provides a more accurate description of the experience of Chinese immigrant adolescents.

The reconceptualization of the adolescent development experience from the perspectives of culture and migration will encourage theoreticians and practitioners to question the dominant view of adolescent development. This study documents the diversity of modes of human development, deconstructs the prevalent Western views of adolescent development, pinpoints the underlying ideological differences between two cultures, and highlight the ways in which the construction of adolescent development is intricately related to the values and ways of thinking. By increasing our understanding of the process of adolescent development in immigrant families, and pointing to the inadequacy of the traditional theories, the study suggests the need to reformulate existing theories of adolescent development.

The results reveal a Chinese conception of adolescent development that is based on the notion that there is a dynamic interplay between culture and migration in immigrant families. In the specific migration context, the indigenous Chinese concepts of bao, guan, and guanxi are reinforced and intensified, and thus develop meanings and themes to the adolescent development process and outcome. Existing theories of adolescent development have been constructed in the Western historical and ideological context, and have led to the devaluation of experiences and perceptions of other cultures. The reconstruction process that we are attempting seeks to develop a more culturally and contextually sensitive theory of adolescent development. It is hoped that when studies in a greater variety of cultural contexts have been completed, we will have a better understanding of what themes, if any, are universal, and a more comprehensive appreciation of the diverse ways in which adolescence is negotiated in different cultures.

The results of this study have important implications for social work practice. The culture-bound nature of current theories is antagonistic to the values and experiences of culturally different groups, and, as a result, the meaning and behavior of minority clients are often misunderstood and misinterpreted. Based on Eurocentric standards of comparison, minorities have been portrayed as pathological, maladjusted, deviant, or delinquent, and these misconceptions have often led to an unrealistic treatment goal or treatment plan[6,59,60,61,62,63]. The inclusion of cultural factors in the understanding of adolescent development would minimize the “Eurocentric cultural biases”[64, p.70]. The emic perspective and the contextual theoretical framework introduced will encourage and assist social workers move to a more contextual and culturally sensitive practice, focusing on individuals in their particular cultural context. If social work practitioners acquire a greater understanding of their minority clients, it will lead to fairer and more effective assessment and treatment. Further, the implications of the Chinese conception of adolescent development for social work practice may serve as an impetus toward redefining the direction of practice. Some therapists with individualistic orientations have misinterpreted the meaning of “relational” development as “dependent”, “regressive”, “symbiotic”, “deficient”, or “weak”. Therapeutic intervention based on this kind of mistaken labeling is harmful and can interfere with the traditional, acceptable, and adaptive values of the Chinese family. The findings illustrate the need to understand clients’ behavior within their own cultural environment and value system.

A number of limitations should be noted of the present study. First, consistent with the qualitative research design, the findings of this study are time, context, and value bound. The findings and the values discussed cannot be applied across all Chinese culture. Second, since participants were limited to a small number of Hong Kong Chinese-Canadian immigrants with a homogenous background, the findings of this study could only reflect their experience and generalizability of the findings is limited. Third, the sampling criteria confined the research participants to specific group of “middle-class” Chinese immigrants from Hong Kong who shared a similar world view. As a result, there are relatively few “intragroup” variations and it is unlikely that this sample adequately reflects the diversity in all Chinese-Canadian families. Lastly, the personal, in-depth nature of the interview and the voluntary basis of participation may have resulted in attracting a specific group of participants; for instance, only caring parents and well-adjusted adolescents, and if this is so, the study results may be biased. Despite these limitations, the present findings are stimulating, particularly in the contributions that they make to the study of adolescents and families in a migration context.

For future research direction, it would be interesting to conduct a similar study of parents and adolescents in Hong Kong and China, or to further replicate the findings in other Chinese communities. This direction is important because it helps to compare their construction of the dynamics of adolescence and the process of adolescent development with that of the Hong Kong Chinese-Canadians. It is likely that the influence of the traditional indigenous values of family loyalty, reciprocity, and guidance may not be as strong in the Chinese context where they are less threatened by Western cultural values. Such a study would provide more understanding of the effect of culture on the construction of adolescent development and would promote the development of theories that would be useful in guiding social work intervention for immigrant families, and for adolescents in Hong Kong and China.
